# Health and Experiences During the COVID-19 Pandemic Among Children and Young People: Analysis of Free-Text Responses From the Children and Young People With Long COVID Study

**DOI:** 10.2196/63634

**Published:** 2025-01-28

**Authors:** Natalia K Rojas, Sam Martin, Mario Cortina-Borja, Roz Shafran, Lana Fox-Smith, Terence Stephenson, Brian C F Ching, Anaïs d'Oelsnitz, Tom Norris, Yue Xu, Kelsey McOwat, Emma Dalrymple, Isobel Heyman, Tamsin Ford, Trudie Chalder, Ruth Simmons, CLoCk Consortium, Snehal M Pinto Pereira

**Affiliations:** 1 Division of Surgery & Interventional Science Faculty of Medical Sciences University College London London United Kingdom; 2 UCL Great Ormond Street Institute of Child Health London United Kingdom; 3 Department of Child and Adolescent Psychiatry Institute of Psychiatry, Psychology & Neuroscience King’s College London London United Kingdom; 4 Division of Psychology and Language Sciences Faculty of Brain Science University College London London United Kingdom; 5 Immunisation Department UK Health Security Agency London United Kingdom; 6 Department of Psychiatry University of Cambridge Cambridge United Kingdom; 7 Department of Psychological Medicine Institute of Psychiatry, Psychology and Neuroscience King’s College London London United Kingdom; 8 See Authors’ Contributions

**Keywords:** children and young people, text mining, free-text responses, experiences, COVID-19, long COVID, InfraNodus, sentiment analysis, discourse analysis, AI, artificial intelligence

## Abstract

**Background:**

The literature is equivocal as to whether the predicted negative mental health impact of the COVID-19 pandemic came to fruition. Some quantitative studies report increased emotional problems and depression; others report improved mental health and well-being. Qualitative explorations reveal heterogeneity, with themes ranging from feelings of loss to growth and development.

**Objective:**

This study aims to analyze free-text responses from children and young people participating in the Children and Young People With Long COVID study to get a clearer understanding of how young people were feeling during the pandemic.

**Methods:**

A total of 8224 free-text responses from children and young people were analyzed using InfraNodus, an artificial intelligence–powered text network analysis tool, to determine the most prevalent topics. A random subsample of 411 (5%) of the 8224 responses underwent a manual sentiment analysis; this was reweighted to represent the general population of children and young people in England.

**Results:**

Experiences fell into 6 main overlapping topical clusters: *school*, *examination stress*, *mental health*, *emotional impact of the pandemic*, *social and family support*, and *physical health* *(including COVID-19 symptoms)*. Sentiment analysis showed that statements were largely negative (314/411, 76.4%), with a small proportion being positive (57/411, 13.9%). Those reporting negative sentiment were mostly female (227/314, 72.3%), while those reporting positive sentiment were mostly older (170/314, 54.1%). There were significant observed associations between sentiment and COVID-19 status as well as sex (*P*=.001 and *P*<.001, respectively) such that the majority of the responses, regardless of COVID-19 status or sex, were negative; for example, 84.1% (227/270) of the responses from female individuals and 61.7% (87/141) of those from male individuals were negative. There were no observed associations between sentiment and all other examined demographics. The results were broadly similar when reweighted to the general population of children and young people in England: 78.52% (negative), 13.23% (positive), and 8.24% (neutral).

**Conclusions:**

We used InfraNodus to analyze free-text responses from a large sample of children and young people. The majority of responses (314/411, 76.4%) were negative, and many of the children and young people reported experiencing distress across a range of domains related to school, social situations, and mental health. Our findings add to the literature, highlighting the importance of specific considerations for children and young people when responding to national emergencies.

## Introduction

### Background

The emergence and rapid spread of SARS-CoV-2 thoroughly disrupted day-to-day life globally, whether through the direct effects of infection or the implementation of preventive control measures [1]. Adolescence is a sensitive period when friendships and social interactions are important for mental health, brain development, and self-concept construction [2]. Thus, the COVID-19 pandemic was a fertile ground for the development and exacerbation of concerns related to children and young people and their mental health, with experts anticipating that it would lead to a higher prevalence of mental health conditions and increased demand on services [3].

In England, COVID-19 preventive measures comprised school closures (with a transition to web-based learning), lockdowns, and restrictions on leisure activities. These restrictions encompassed limits on time spent outdoors, as well as regulations on whom individuals could interact with and how they did so (ie, social distancing). The implementation of such measures was feared to be detrimental to the mental health of children and young people [4]; for example, the effects of school closures and social distancing, including their resultant loneliness, can be long-lasting, with evidence from previous pandemics suggesting that associations with poorer mental health can persist for nearly a decade [5]. Indeed, pandemics can exert a broad negative impact on children and young people, causing stress and feelings of helplessness, as well as behavioral, sleep, and eating problems, with higher rates of depression and anxiety likely during and after a pandemic [6].

Regarding the COVID-19 pandemic specifically, the literature is equivocal as to whether the predicted deterioration in mental health came to fruition. Some quantitative studies report more children and young people reaching thresholds for mental health conditions [7] and increased emotional problems and depression [4,8]. However, other studies report improved mental health and well-being, including sleep hygiene [9] and fewer externalizing and internalizing problems [10-12]. Therefore, it is unsurprising that a systematic review of 51 studies revealed heterogeneity regarding the impact of COVID-19 on the mental health of children and young people [12]. Similarly, qualitative explorations of the experiences of children and young people during the pandemic revealed heterogeneity, with prominent themes ranging from feelings of loss to growth and development [13]; for example, while most children and young people adapted well to school closures and web-based learning, experiences ranged from a lack of motivation to complete work to improved productivity [14]. Equally, while some children and young people from Black and ethnic minority backgrounds reported a negative mental health impact, others reported improved well-being and better coping strategies after lockdown [15]. More broadly, many children and young people aged 13 to 24 years across the United Kingdom reported worse mental health, school-related concerns, and socializing concerns, while a minority viewed pandemic experiences and the resultant changes as helpful (eg, strengthened familial relationships) [16]. Reasons for the observed heterogeneity are multifactorial and could include a research focus on specific subpopulations of children and young people (eg, those with special educational needs [17], specific ethnic groups [15], or groups considered vulnerable and high risk [7,11]) rather than the general population of children and young people; alternatively, experiences of children and young people could vary by pandemic stage [18]. Indeed, a lack of generalizability was a limitation highlighted in previous studies with small samples (eg, N=168 [4] and N=37 [16]) recruited from specific regions [4,8-11,14,15,19]. Similarly, qualitative research draws on the experiences and beliefs of small, selective samples and can rarely be considered to provide generalizable conclusions (although generalizability is not the primary aim of such research) [20].

### Objectives

To overcome this limitation, we analyzed 8224 self-reported free-text responses from children and young people. Specifically, we used free-text responses from the Children and Young People With Long COVID (CLoCk) study, which recruited >30,000 children and young people aged 11 to 17 years when they underwent polymerase chain reaction (PCR) testing for SARS-CoV-2 between September 2020 and March 2021 [21]. At recruitment, children and young people were asked “to tell us about [their] health or how the pandemic or lockdown affected [them].” We aimed to analyze these responses to determine the most important topics and the main “sentiment” to understand the impact of the pandemic. To ensure that our findings were representative of the broader population of children and young people in England, we applied survey weights [22] to the sentiment analysis.

## Methods

### Study Design and Setting

In this study, we focus on the free-text responses of children and young people (aged 11 to 17 y at index PCR test) collected at study enrollment, which was 3, 6, or 12 months after their index PCR test (ie, April 2021-December 2021). During this period, most pandemic-related restrictions were gradually lifted (April-July 2021), until the beginning of December 2021 when measures were reimplemented to slow the spread of the SARS-CoV-2 Omicron variant [23].

The UK Health Security Agency’s Second Generation Surveillance System, which is a dataset containing results of all SARS-CoV-2 PCR tests conducted by hospital and public health laboratories as part of national mandatory testing, was used to identify 219,175 children and young people (n=91,014, 41.53% tested positive, while n=128,161, 58.47% tested negative) who underwent PCR testing between September 2020 and March 2021 [21,24]. Children and young people who tested positive were matched, at CLoCk study invitation, to those who tested negative based on age, sex, region of residence, and month of test. Index of Multiple Deprivation (a proxy for socioeconomic status, grouped into quintiles) was determined via the lower super output area (ie, small local area level–based geographic hierarchy) in which the children and young people resided [25]. Consenting children and young people completed web-based questionnaires 3, 6, 12, and 24 months after their index PCR test, although the sweeps of data collection varied by month of test [21,24]; for example, children and young people who tested in January to March 2021 were able to provide data 3, 6, 12, and 24 months after the test compared to those who tested in October to December 2020 who were only able to do this at 6, 12, and 24 months. The questionnaire included demographics, elements of the International Severe Acute Respiratory and Emerging Infection Consortium pediatric COVID-19 questionnaire, and several validated scales (eg, the Strengths and Difficulties Questionnaire [26]). The questionnaire ended with a final, optional, free-text response question prompting children and young people to “Please use this space if there is anything else you would like to tell us about your health or how the pandemic or lockdown have affected you.” This was the final question before survey submission. This study focused solely on the free-text responses provided at study enrollment.

### Participants and Sample Size

**Figure 1:**
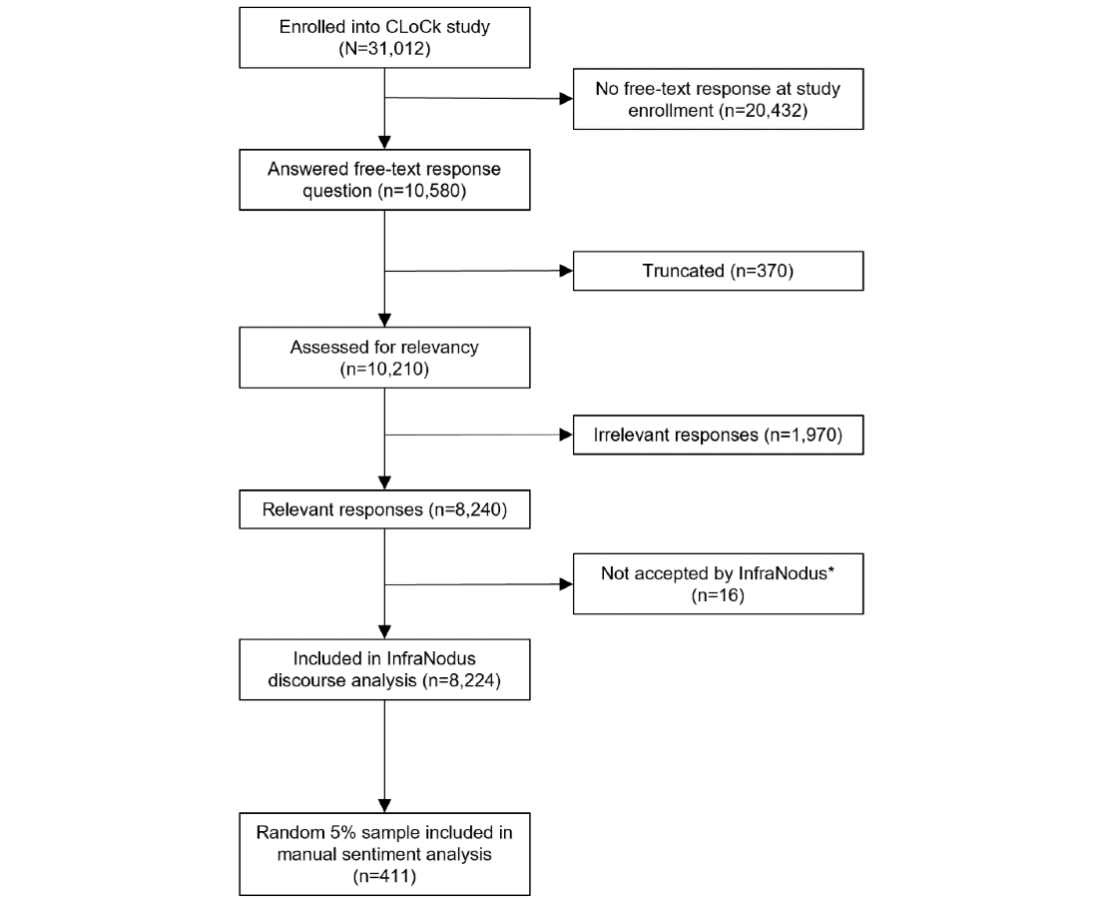
Participant selection flowchart. CLoCk: Children and Young People With Long COVID.

In total, 31,012 children and young people enrolled into the CLoCk study [21] (ie, 31,012 children and young people submitted their enrollment questionnaire). Of these 31,012 respondents, 10,580 (34.12%) completed the free-text response question (Figure 1). The web-based questionnaire platform truncated free-text responses at 621 characters; to ensure that the experiences of the children and young people were not misrepresented, we excluded from analysis all responses exceeding 619 characters (370/10,580, 3.5%). Thus, 10,210 (96.5%) of the 10,580 responses were subject to data cleaning and relevancy assessment as described in the next subsection.

### Data Cleaning and Relevancy Assessment

Free-text responses were fully anonymized—any potentially identifiable information (eg, child’s or young person’s name, email addresses, and hospital names) was redacted; for example, an anonymized response was as follows:

[T]he only thing that affected me is the move from [city name] to [city name] but regarding covid my mental health is ok although during the lockdown it was often low.

All 10,210 responses were assessed for relevancy (refer to Textbox 1; of note, statements regarding COVID-19 vaccination, its immediate symptoms, timing with regard to SARS-CoV-2 infection or both were excluded because we are interested in the effects of SARS-CoV-2 itself and the associated COVID-19 preventive measures, eg, lockdowns, rather than vaccination side effects; moreover, the exclusion criteria are not mutually exclusive: a response may have been excluded for multiple reasons). One author (NKR) screened all responses. Any uncertainties were discussed with another author (SMPP) to reduce subjectivity and potential bias. Of the 10,210 responses assessed for relevancy, 1528 (15%) were discussed. Certain words or phrases were standardized across responses for consistency; for example, all variations of “COVID-19” (eg, “covid-19,” “COVID 19,” and “covid19”) were revised to read “COVID.” The final stage of data cleaning involved the checking and correction of spelling mistakes by 3 authors (NKR, LFS, and AD), with any uncertainties on how to rectify a spelling mistake (33/8240, 0.4%) discussed to achieve consensus.

**Relevancy assessment criteria and selected examples of free-text responses (included and excluded) provided by children and young people and not truncated at study enrollment (n=10,210).**

**Inclusion criteria (included: n=8240)**

- Statements about the respondents’ health related to SARS-CoV-2 infection, their health during the COVID-19 pandemic more generally or both.
- Statements about the respondents’ experience of the pandemic, lockdown or both.
- Examples
  - “During the pandemic, I did lose quite a few friends and my mental health got worse”
  - “I enjoyed online learning as it gave me the opportunity to self teach something I found I am good at and I feel it is a useful skill for development. I was fitter during lockdown as I had more time to exercise and many good traits I picked up in lockdown I have kept”
  - “The first lockdown triggered phobias and anxiety. This worsened after grandad was in hospital with COVID”

**Exclusion criteria (excluded: n=1970)**

- Null responses (eg, “No,” “N/A,” or equivalent)
- Statements unrelated to the respondents’ health or experiences of the pandemic or lockdown
- Nonsensical responses
- Statements revealing diagnoses before the pandemic (eg, autism)
- Statements discussing medication, health, or experiences before the pandemic with no comparison to medication, health, or experiences during the pandemic
- Statements about COVID-19 vaccination: its immediate symptoms and timing with regard to SARS-CoV-2 infection
- Responses related to whether the respondents tested positive for SARS-CoV-2 and its immediate impact (eg, on cough or smell within the first few weeks after infection)
- Statements on health guidance (eg, opinions on vaccination strategy) with no reference to their impact on the respondents personally
- Current colds, coughs, influenza, hay fever, seasonal allergies, and so on
- Correcting or clarifying previous questionnaire entries and expressing dissatisfaction with survey
- Examples
  - “TEST”
  - “are we going to go into another lockdown?”
  - “icciycih”
  - “I called [footballer] for my birthday, it was a small party. Suddenly, he arrived at the restaurant with a box of chocolates, all ashamed. He apologised for the gift and said he didn’t know what to get because he had never been invited to a birthday party before”
  - “Have chronic fatigue syndrome (ME) since before COVID 19”
  - “I am allergic to aspirin”
  - “I have had glandular fever so most of these problems are and can be linked to it”
  - “I have had two Pfizer vaccinations, with an achy arm for a day or two as my only symptoms”
  - “China made covid Please tell boris that I want to go to him next party”
  - “Boris Johnson is rude”
  - “Help the NHS!! No more pandemic and vaccine effort until they have sufficient support in my opinion”

### Quantitative Text Analysis

InfraNodus, an artificial intelligence (AI)–powered tool that processes and visualizes text-based data [27], was used. The tool follows these steps [28]:

1. Text normalization: words are converted into their lemmas (ie, the dictionary form) to reduce redundancy (eg, “families” becomes “family”), and syntax elements (eg, commas) are removed.
2. Stop word removal: words without significant semantic meaning (eg, “is” and “are”) are filtered out. InfraNodus generates a default stop word list based on the uploaded text, which can be customized (see InfraNodus Discourse Analysis section).
3. Network graph visualization: words are displayed as a network graph that highlights the most influential words, with the option to detect topical clusters (ie, groups of words that tend to co-occur). The network graph is interactive, allowing users to read through the text by relevant concepts (vs reading in a linear fashion).

### InfraNodus Discourse Analysis

The terms “health,” “COVID,” “pandemic,” and “lockdown” were part of the question wording. Therefore, before analysis, these words were included as stop words because they could potentially conceal other important topics. InfraNodus was then used to determine the most influential words and measure patterns occurring within the relevant 8224 free-text responses examining the experiences and health during the pandemic of children and young people. This was achieved using “betweenness centrality” scores [28]. This is a numerical measure of a word’s influence over information flow and its ability to link different parts of the text network graph [28]. Words with the highest betweenness centrality scores were represented as larger nodes on the graph. Subsequently, topic modeling was undertaken. To achieve this, InfraNodus applied a community detection algorithm that identified groups of words more densely connected to each other than to the rest of the network, yielding an output of topical clusters [28].

### Sentiment Analysis

Sentiment analysis measured the positive, negative, or neutral feelings reported by children and young people in the free-text responses. Initially, we set out to use InfraNodus for the sentiment analysis of all eligible responses; however, there were major discrepancies between researcher-assigned and InfraNodus-assigned sentiment (refer to Table S1 in Multimedia Appendix 1 for examples). Thus, to assess the main “sentiment” of children and young people during the COVID-19 pandemic, sentiment analysis was undertaken manually using the sentiment framework outlined in Textbox 2. A random sample was chosen for the sentiment analysis for pragmatic reasons, given the time available. This sample size represented 5% (411/8224) of the available data. The random selection aimed to mitigate potential biases inherent in other selection approaches, such as sequential sampling, which may have resulted in selection of responses predominantly from a specific time point. NKR and a second rater (LFS) met to discuss in depth the sentiment framework presented in Textbox 2, going through examples of positive, negative, and neutral statements. Subsequently, NKR’s rater reliability was assessed by LFS who assigned a sentiment to a random subsample of 206 (50.1%) of the 411 responses. Agreement between the raters was 94.7% (195/206). Chi-square tests were used to examine associations between free-text sentiments and demographics.

**Sentiment framework for classification of free-text responses provided by children and young people at study enrollment.**

**Positive sentiment**

- Largely communicating relatively high or high levels of well-being during the pandemic
- Largely communicating satisfaction with health guidelines and COVID-19 preventive measures (eg, school closures and lockdowns) and associated outcomes
- Largely communicating good health during the pandemic
- Examples
  - “I think the pandemic and lockdown has motivated me to be healthier and I am in overall better mental and physical health than before the pandemic”
  - “I liked being at home and doing schoolwork at home. But I missed seeing people”
  - “I loved the lie ins”

**Neutral sentiment**

- No positive or negative expressions
- General, factual statements
- Examples
  - “Made me be more independent- online learning and having to study the curriculum for my GCSE exams in 2021”
  - “Gained some weight”
  - “I live in an SGO I have not been able to see my mum as she lives in a hostel for over a year”

**Negative sentiment**

- Largely communicating poor well-being during the pandemic, including concerns about family members’ health
- Largely communicating upset, frustration, or concern with health guidelines or COVID-19 preventive measures (eg, school closures and lockdowns) and associated outcomes
- Largely communicating poor health
- Examples
  - “I have not been able to see my friends and feel lonely. It scares me and makes me anxious, I have trouble concentrating”
  - “Found it very hard to stay on top of schoolwork so it has affected my mental health as I am quite stressed”
  - “I feel that my education has suffered because of lockdowns and within lockdowns I have gained problems with eating.”

### Reweighting: Sentiment Analysis

Sentiment analysis reweighting was conducted using Stata 17 (StataCorp LLC) [29]. Multimedia Appendix 1 provides detailed information on survey weight development and application, which is briefly summarized herein. Survey weights were developed to account for (1) not all children and young people invited to participate enrolling into the study, (2) not all children and young people enrolling providing a free text response, (3) not all free text respondents having done so relevantly (including within the character limit), and (4) not all those responding relevantly being randomly sampled for manual sentiment analysis. These 4 developed survey weights were combined to obtain a fifth survey weight that was used to reweight the analytic sample (n=411) to the target population (ie, all those invited to enroll; N*=*219,145). As the target population may not be representative of children and young people in England [22], we recalibrated our sentiment analysis to the general population of children and young people in England using census data from 2021 [30].

### Ethical Considerations

Research ethics approval was granted by the Yorkshire and The Humber—South Yorkshire Research Ethics Committee (21/YH/0060; Integrated Research Application System project ID: 293495). Web-based informed consent was obtained from parents or carers of children and young people aged ≤15 years, with these children and young people also providing web-based assent. Those aged 16 to 17 years provided web-based informed consent, but their parents or carers did not, as per UK Health Research Authority–recommended processes [31]. Data privacy and confidentiality were maintained by redacting any potentially identifiable information in the free text (eg, child’s or young person’s name, email addresses, and hospital names) before analysis to achieve full anonymization. Those who completed and submitted questionnaires were compensated with a £10 (US $12.23) voucher for each completed questionnaire.

## Results

### Participant Demographics

Compared to those invited to participate in the CLoCk study, those included in the InfraNodus and sentiment analyses were broadly similar, with, for example, participants more likely to be female individuals (Table 1). There were some demographic differences in those excluded due to truncation and irrelevancy; for example, while more female individuals (279/370, 75.4%) and older children and young people (253/370, 68.4%) were excluded due to truncation, younger children and young people (1088/1970, 55.2%) were more likely excluded due to irrelevancy (Table S2 in Multimedia Appendix 1).

**Table 1:** Demographics of children and young people who (1) were invited, (2) enrolled, (3) answered the free-text question at enrollment, (4) were included in the InfraNodus analysis, and (5) were included in the manual sentiment analysis.

		Invited (N=219,175)	Enrolled (n=31,012)	Answered free-text question (n=10,580)	Included in InfraNodus analysis (n=8224)	Included in sentiment analysis (n=411)
**COVID-19 status at study invitation**						
	SARS-CoV-2 positive	91,014 (41.53)	13,690 (44.14)	4743 (44.83)	3804 (46.25)	180 (43.8)
	SARS-CoV-2 negative	128,161 (58.47)	17,322 (55.86)	5837 (55.17)	4420 (53.75)	231 (56.2)
**Sex**						
	Male	103,939 (47.42)	11,961 (38.57)	3920 (37.05)	2905 (35.32)	141 (34.31)
	Female	115,236 (52.58)	19,051 (61.43)	6660 (62.95)	5219 (64.68)	270 (65.69)
**Age (y) at study invitation**						
	11-14	112,057 (51.13)	14,857 (47.91)	5184 (49)	3973 (48.31)	189 (45.99)
	15-17	107,118 (48.87)	16,155 (52.09)	5396 (51)	4251 (51.69)	222 (54.01)
**Ethnicity**						
	Asian or Asian British	—^a^	4553 (14.68)	1581 (14.94)	1143 (13.9)	60 (14.6)
	Black British, African British, or Caribbean British	—	933 (3.01)	343 (3.24)	264 (3.21)	18 (4.38)
	White	—	23,198 (74.8)	7838 (74.08)	6167 (74.99)	308 (74.94)
	Mixed	—	1615 (5.21)	564 (5.33)	464 (5.64)	21 (5.11)
	Other	—	524 (1.69)	187 (1.77)	135 (1.64)	1 (0.24)
	Prefer not to say	—	189 (0.61)	67 (0.63)	51 (0.62)	3 (0.73)
**Region of residence (England)**						
	East Midlands	14,109 (6.44)	2210 (7.13)	774 (7.32)	601 (7.31)	30 (7.3)
	East of England	38,901 (17.75)	6047 (19.5)	1967 (18.59)	1543 (18.76)	63 (15.33)
	London	46,300 (21.12)	6157 (19.85)	2107 (19.91)	1565 (19.03)	72 (17.52)
	North East England	8613 (3.93)	1198 (3.86)	403 (3.81)	329 (4)	18 (4.38)
	North West England	31,289 (14.28)	3606 (11.63)	1229 (11.62)	966 (11.75)	49 (11.92)
	South East England	31,567 (14.40)	4917 (15.86)	1729 (16.34)	1347 (16.38)	80 (19.46)
	South West England	8139 (3.71)	1514 (4.88)	499 (4.72)	396 (4.82)	25 (6.08)
	West Midlands	22,681 (10.35)	3032 (9.78)	1046 (9.89)	822 (10)	37 (9)
	Yorkshire and the Humber	17,576 (8.02)	2331 (7.52)	826 (7.81)	655 (7.96)	37 (9)
**IMD^b^ quintile**						
	1 (most deprived)	54,079 (24.67)	5345 (17.24)	1813 (17.14)	1397 (16.99)	74 (18)
	2	44,757 (20.42)	5548 (17.89)	1943 (18.36)	1484 (18.04)	80 (19.46)
	3	39,876 (18.19)	5792 (18.68)	1985 (18.76)	1563 (19.01)	80 (19.46)
	4	39,996 (18.25)	6656 (21.46)	2220 (20.98)	1764 (21.45)	91 (22.14)
	5 (least deprived)	40,467 (18.46)	7671 (24.74)	2619 (24.75)	2016 (24.51)	86 (20.92)

^a^Ethnicity data were not available for children and young people at study invitation; this was self-reported at study enrollment.

^b^IMD: Index of Multiple Deprivation.

### InfraNodus Discourse Analysis

The InfraNodus discourse analysis revealed 6 main topical clusters related to *school*, *examination stress*, *mental health*, *emotional impact of the pandemic*, *COVID-19 symptoms*, and *social and family support*. Each cluster is described in detail in the following subsections (of note, the majority of statements fell into multiple clusters).

#### School

Children and young people reported struggling academically, with participants stating that the pandemic “affected my school learning a lot” (PID 8126), and that absence due to restrictions caused much stress, upset, and worry due to finding it “difficult to catch up with work I have missed” (PID 7995). Concentration was commonly reported as an issue, with a respondent reporting having found “it harder to concentrate in class, harder to complete homework without being distracted and more stressful to complete tests” (PID 6851). However, there were some children and young people who enjoyed web-based classes during COVID-19 restrictions, even going as far as to say, “I liked online school” (PID 8161) and “I prefer home schooling” (PID 8196). Nevertheless, others struggled, commenting as follows: “I...hated having to do my work on a laptop” (PID 8076) and “I cannot concentrate as well as in the classroom” (PID 8051). Moreover, social skills seem to have been affected by school closures, with a participant stating that they “find it difficult to communicate with people and [I] get uncomfortable when people approach [me]” (PID 8146), while another shared as follows:

I feel it negatively affected my social skills slightly, making it harder for me to talk to acquaintances.

PID 8046

#### Examination Stress

In this cluster, children and young people reported high levels of stress due to COVID-19 restrictions and their impact on examinations. One young person reported as follows:

[S]chool is way more stressful, there’s so many changes to how exams are going or if we don’t have any etc. such as what subjects are being dropped or topics we don’t need etc.

PID 7531

Another person berated the government, noting that “the way the government dealt with my GCSEs was very stressful” (PID 6101). A common complaint was the government’s handling of examinations, with this varying depending on pandemic stage; for example, some had qualms with the sudden news of an exam:

[D]on’t tell people they are not going to have exams and then give them exams with less than a month’s warning. It’s honestly a joke.

PID 5386

Others struggled with the consequences of GCSEs being cancelled:

As my GCSEs were obviously cancelled I have been sitting up to 5 exams every day for weeks.

PID 5996

Some expressed frustration at “all that study [being] wasted” (PID 4891) and even attributed the physical symptoms felt (eg, tiredness and weakness) to the “uncertainty around my exams” (PID 5996).

#### Mental Health

Children and young people became more aware of their mental health during the pandemic, with a respondent stating, “I think I have some mental illnesses” (PID 8221) and another sharing that “the lockdowns have severely affected my mental health” (PID 8176). Many children and young people reported having ≥1 mental health conditions (eg, oppositional defiant disorder, obsessive-compulsive disorder, eating disorders, and depression) and commented on the impact that COVID-19 preventive measures had on them:

What affected me the most was having to be in and out of school when there was a case of coronavirus as I find it hard to go to school and this made my anxiety disorder worse as I had to restart going back after two weeks of isolation.

PID 8096

Nevertheless, some children and young people reported that their “mental health got better” (PID 5629), while one respondent commented as follows:

[T]he pandemic helped me to focus on my mental health and forced me to better look after myself so, in a way, it was positive for me.

PID 7716

#### Emotional Impact of the Pandemic

Anxiety was a common complaint:

I have felt very anxious about COVID. It has made me not want to leave my house or parents in case they die.

PID 51

[T]he pandemic makes me more worried in general about the small things in everyday life.

PID 31

Similarly, a respondent expressed being “a bit more worried about the future and also what may happen as a direct impact of the pandemic” (PID 346). Many children and young people reported experiencing frustration, feeling unhappy, and being more prone to worrying during the pandemic. Moreover, loneliness was also a common complaint, with a respondent reporting as follows:

[T]he lockdown and the pandemic have made me feel a lot more isolated than I previously did.

PID 291

#### Physical Health (Including COVID-19 Symptoms)

Within this topical cluster, the children and young people mainly reported the long-lasting impact of testing positive for COVID-19 infection, with changes to smell and taste being prominent:

The fact that food tastes and smells bad in some cases (smells bad in many cases) has impacted how much I eat and my diet, for instance I can’t eat eggs anymore as they taste rotten. My diet has changed as a result.

PID 46

I have not been able to taste very well for about the past 3 months ever since I got COVID. This includes foods tasting very weird, not eating food I used to like, not being able to smell clear things other people can.

PID 196

The impact of changes to smell and taste seemed to be long-lasting; for example, a respondent stated they got their “taste partially back 11 months after I was positive for coronavirus” (PID 2451). Some children and young people made a reference to the term “long COVID”:

I have long COVID...where I have distorted/lost my smell and taste.

PID 1621

Sleep disturbances were also commonly reported:

“[M]y sleeping pattern started to go downhill. I was awake all night and sleeping through the day.

PID 861

However, there were some who did not report such difficulties, stating they “only had minor symptoms which went away within a week” (PID 1851) or that they “don’t feel I have had any long term issues as a result of having COVID” (PID 2786).

#### Social and Family Support

There was marked heterogeneity in terms of the impact of COVID-19 on social and family support. Some children and young people reported feeling distanced:

During COVID I didn’t have as much contact with friends or family so I became a bit distant.

PID 736

It affected me as I couldn’t hang out with my friends and I feel like I’ve lost a connection between them.

PID 2251

Others shared the opposite experience:

COVID has been devastating for everyone. It’s a very difficult and challenging time. However, it has helped me become closer with my family and friends.

PID 756

Some made use of web-based platforms to engage with their friends (eg, game consoles and Microsoft Teams), which helped mitigate the impact of the COVID-19 preventive measures. A respondent reported having “been able to spend a lot of time with my friends virtually using my games console so haven’t felt bored or lonely” (PID 1016). Though children and young people acknowledged the impact of the lockdown in relation to loss of support:

[M]y support system of friends were suddenly cut off and couldn’t be there physically for me anymore.

PID 1026

However, some also described how it expanded social circles:

[O]ver the course of the pandemic I will say that I have made more friends than I would have otherwise because people would add me and my friends into group chats. Some of my closest friends have come from this.

PID 1026

Familial problems were reported by some children and young people, with a respondent stating that they “argued with my parents because I spend all my time with them” (PID 3676). Another stated as follows:

I think the pandemic has affected me badly. Being confined in a space with my mum and sister and not having a personal space to debrief has been difficult. I have realised a lot about myself and my family and they’re not good things but I am grateful that I know this information otherwise I would have gone living my life like I was wrong all the time.

PID 3271

### Sentiment Analysis: Original and Reweighted

Manual sentiment analysis of the 411 randomly sampled free-text responses yielded 314 (76.4%) negative, 57 (13.87%) positive, and 40 (9.73%) neutral statements. There were observed associations between the free-text sentiment and COVID-19 status as well as sex (*P*=.001 and *P*<.001, respectively; Table S3 in Multimedia Appendix 1) such that most of the responses, regardless of COVID-19 status or sex, were negative; for example, 84.1% (227/270) of the responses from female individuals and 61.7% (87/141) of those from male individuals were negative (Table S3 in Multimedia Appendix 1). There were no observed associations between the free-text sentiment and all other examined demographics (ie, age, ethnicity, region of residence, and Index of Multiple Deprivation quintile). When reweighted to the general population of children and young people in England, sentiment proportions remained largely consistent: 78.52% (negative), 13.23% (positive), and 8.24% (neutral). Most of those with negative sentiment were female (227/314, 72.3%), while those with positive sentiment were mostly older (35/57, 61%; Table 2).

**Table 2:** Unweighted and reweighted demographics of the randomly sampled children and young people stratified by the sentiment of the free-text response provided at study enrollment (n=411).

			Positive sentiment (n=57)				Neutral sentiment (n=40)				Negative sentiment (n=314)		
			Observed (%)		Reweighted (%)		Observed (%)		Reweighted (%)		Observed (%)		Reweighted (%)
**COVID-19 status at index** **PCR^a^ test**													
	SARS-CoV-2 positive	13.33		13.52		3.89		3.71		82.78		82.77	
	SARS-CoV-2 negative	14.29		12.96		14.29		12.58		71.43		74.46	
**Sex**													
	Male	21.28		20.5		17.02		13.84		61.7		65.66	
	Female	10		9.13		5.93		5.08		84.07		85.79	
**Age (y) at study invitation**													
	11-14	11.64		11.32		12.17		10.04		76.19		78.63	
	15-17	15.77		14.86		7.66		6.7		76.58		78.43	
**Ethnicity**													
	Asian or Asian British	18.33		15.57		10		11.38		71.67		73.05	
	Black British, African British, or Caribbean British	16.67		13.06		0		0		83.33		86.94	
	White	12.01		12.13		10.71		8.85		77.27		79.01	
	Mixed	28.57		24.83		4.76		2.31		66.67		72.86	
	Other	0		0		0		0		100		100	
	Prefer not to say	0		0		0		0		100		100	
**Region of residence (England)**													
	East Midlands	6.67		6.49		13.33		11.85		80		81.66	
	East of England	11.11		14.02		11.11		11.54		77.78		74.44	
	London	19.44		20.29		12.5		11.58		68.06		68.13	
	North East England	5.56		4.71		16.67		13.17		77.78		82.12	
	North West England	4.08		5.82		6.12		4.78		89.8		89.4	
	South East England	22.5		17.97		10		8.46		67.5		73.57	
	South West England	16		16.81		4		2.87		80		80.32	
	West Midlands	16.22		15.91		2.7		2.72		81.08		81.37	
	Yorkshire and the Humber	8.11		8.11		10.81		9.87		81.08		82.01	
**IMD^b^ quintile**													
	1 (most deprived)	14.86		12.66		8.11		6.33		77.03		81.01	
	2	17.5		16.35		11.25		10.35		71.25		73.3	
	3	6.25		4.94		10		6.44		83.75		88.63	
	4	10.99		14.08		6.59		7.24		82.42		78.68	
	5 (least deprived)	19.77		19.1		12.79		11.37		67.44		69.53	

^a^PCR: polymerase chain reaction.

^b^IMD: Index of Multiple Deprivation.

## Discussion

### Principal Findings

Using an innovative AI method and self-reported data from 8224 children and young people during the COVID-19 pandemic, we found that the experiences of the children and young people fell into 6 main topical clusters related to *school*, *examination stress*, *mental health*, *emotional impact of the pandemic*, *physical health (including COVID-19 symptoms)*, and *social and family support*. Importantly, there was substantial overlap between the topical clusters, such that, for example, *examination stress* and *school* could be collapsed into 1 cluster. Similarly, *mental health* and *emotional impact of the pandemic* could be classified together under *well-being*. Manual sentiment analysis showed that the majority of statements made by children and young people were largely negative (314/411, 76.4%), with only a small proportion (57/411, 13.9%) classified as positive. When reweighted to the general population of children and young people in England, the overriding sentiment prevalences were largely unchanged.

Our findings are in line with previous literature using different methods that saw COVID-19–related school closure as a contributing factor to the increased stress, frustration, anxiety, and loneliness experienced by children and young people [32]. Repeated cross-sectional comparisons (before and after the pandemic) of children and young people referred to specialist Child and Adolescent Mental Health Services found an increase in emotional problems and depression as well as the number of children and young people reaching thresholds for mental health conditions in those recruited at the time of schools reopening [7]. The authors suggest that this may be due to stresses involved in adjusting to the return to school. Qualitative descriptions paint a similar picture, with a minority benefiting from school closures (eg, because of reduced stress due to the lack of examinations), while the majority wanted to return for the social aspect and reported concentration difficulties, fears of falling behind, and frustration at their seemingly fruitless efforts [33]. Likewise, the examination by Scott et al [34] of the diary entries of children and young people over a 6-week period between July and October 2020 revealed that the pandemic had a negative impact on the mental health of children and young people, with many reporting anxiety, loss, stress, and loneliness, which is in line with the mental health and emotional impact of the pandemic clusters found in this study. Similar to our findings, while concerns surrounding school-related matters were prevalent (eg, long-term consequences of school closures and cancelled examinations), a minority of children and young people viewed school closures positively [34]. Sleep disturbances and loss of taste and smell commonly reported within the topical cluster of COVID-19 symptoms were in line with previous findings, with sleep hygiene negatively affected [11]. In terms of social and family support, the loneliness reported by some children and young people within this cluster is in line with quantitative data from the CLoCk study [23,35]. More broadly, previous literature indicates that 27% and 8% of children and young people reported “slightly more” and “much more” loneliness, respectively, during the first UK national lockdown [19].

The heterogeneity highlighted in this study brings together seemingly opposing findings in the literature; for example, some previous studies reported improved familial and peer relationships [9], whereas others reported tensions at home [36]. An explanation for previous discrepant findings and our current heterogeneous results may be explained by noting that variation in health and well-being by pandemic stage is possible; for example, in the Co-SPACE study, trends in emotional, conduct, and hyperactivity problems followed changes in COVID-19 preventive measures, that is, most problems increased after lockdown announcements and decreased as restrictions eased and schools reopened [18]. Notably, studies reporting improved mental health were typically conducted during the first UK national lockdown (March 2020-June 2020), which was novel because it was children’s and young people’s first experience of a lockdown and may have been qualitatively different from subsequent lockdowns [9]. Using data that largely come from children and young people themselves allows for their voices to be heard both in terms of the content of their written expressions and also the sentiment of the words used. Although previous studies have indicated the heterogeneity of experiences [37], this is the first study that used sentiment analysis to quantify the heterogeneity of experiences and health of children and young people during the pandemic. We found that more than three-quarters of the written expressions of children and young people were negative (314/411, 76.4%), and most of these negative statements were written by female individuals (227/314, 72.3%). By contrast, <15% (57/411) of the statements were positive, and these were mostly written by older children and young people (35/57, 61%). Understanding the basis for the heterogeneity of experiences and health during the pandemic is important because it helps identify factors that may contribute to resilience in the face of adversity as in the case of the pandemic. Notwithstanding these findings, it has also been highlighted that psychological distress during the COVID-19 pandemic varied across countries [38], with, for example, a quarter of the children and young people surveyed in Malaysia reporting depressive symptoms during lockdown and amid rising cases and fatalities [39], while 37% of children and young people aged 18 to 24 years in Southeast and South Asia reported positive attitudes toward the pandemic [40]. Thus, our findings might be restricted to children and young people in England.

Our study’s large sample size is a strength, taking into account the responses of >8000 children and young people regarding their experiences during the pandemic. Nonetheless, study limitations are acknowledged; for example, InfraNodus topical clusters were not entirely distinct: *examination stress* and *school* were similar, as were *mental health* and *emotional impact of the pandemic*. While the AI tool we used considered these as discrete clusters, we acknowledge that children and young people are complex beings, and the thoughts and emotions expressed in their written responses could straddle multiple clusters. While manual examination of the responses did not yield substantive differences between some clusters, the AI software did not allow for the identified clusters to be collapsed. Software limitations were further highlighted in the sentiment analysis. We intended to use the AI software to run the sentiment analysis on all 8224 free-text responses. However, our analysis highlighted several notable discrepancies between InfraNodus-assigned and human-assigned sentiment, especially in complex, nuanced statements. Examples of these discrepancies are provided in Table S1 in Multimedia Appendix 1. This issue reflects a limitation in the AI tool’s capacity to capture the full emotional range in the responses, particularly in contexts involving children’s expressions, which may convey implicit distress or complex emotional states not adequately interpreted by keyword-based algorithms. InfraNodus was not specifically trained on data from children and young people, which likely contributed to its misinterpretations. Sentiment analysis tools developed for general audiences often lack the contextual sensitivity needed for younger populations, whose expressions may differ significantly in language complexity, structure, and sentiment cues. This highlights the need for AI sentiment tools to be trained specifically on data from children and young people to improve their interpretive accuracy. Furthermore, while we considered applying a natural language processing approach to extend the themes generated from our sentiment analysis and to predict themes across the full sample, this was not feasible within our current resources. Developing a contextual sentiment analysis framework for this purpose, which would allow for predictive insights and the potential identification of overlooked themes, would have required substantial computational support and additional funding. As such, this remains a limitation of our study. We recommend that future research consider integrating natural language processing methods such as topic modeling to analyze the remaining 95% (7813/8224) of the free-text responses, thereby enhancing theme coverage and providing a more comprehensive understanding of the thematic patterns across all data. In addition, reweighting the discourse analysis would have helped ensure representativeness, although this was not possible with the software used. These limitations highlight the importance of not relying exclusively on AI-generated interpretations of text and the need for specialist, substantive knowledge of the topics and data examined.

A small percentage of responses (370/10,580, 3.5%) were truncated and excluded from analysis. However, as the prevalence of truncation was low, it is unlikely that this exclusion introduced bias into our analysis. More generally, although the children and young people invited to take part in the CLoCk study were a nationally representative sample, they are a specific population (children and young people aged 11-17 y who underwent PCR testing). Therefore, children and young people who are invisible to the public health system may be excluded. In addition, there is further potential for selection bias because only 34.12% (10,580/31,012) of those completing the questionnaire answered the free-text response question. However, there seem to be no major demographic differences between those enrolling and those who answered the free-text question. To mitigate this bias, future research might consider making the free-text response question compulsory. The underrepresentation of positive sentiments may reflect a tendency for those more negatively impacted by the pandemic to respond to both the questionnaire and provide a relevant free-text response. To mitigate these selection issues, the sentiment analysis was reweighted to the general population of children and young people in England using census data from 2021 [30]. Although the questionnaire was designed to be completed by children and young people themselves, there was no way to check that responses were written by the children and young people as opposed to a parent or carer. Moreover, parental influence may have affected responses among those children and young people who completed it with parental assistance. Future studies would benefit from distinguishing between the input of children and young people and parental input by, for example, including a question asking whether the questionnaire was completed solely by the child or young person, solely by a parent or carer, or primarily by the child or young person with assistance from a parent or carer.

As in any manual qualitative data analysis, unconscious rater bias had the potential to affect the results. Thus, measures were taken to minimize its impact: for the manual sentiment analysis, a second rater assessed the sentiment of half the responses (206/411, 50.1%), yielding excellent agreement (195/206, 94.7%) between the raters. We acknowledge that the relevancy assessment of all 10,580 free-text responses by a second rater would have been beneficial; however, this was not possible due to limited capacity. Nevertheless, subjectivity in determining the relevance of statements during the relevancy assessment was mitigated by discussing all uncertainties with another researcher and by adopting a collaborative approach to developing the relevancy criteria. Finally, we acknowledge that temporal variation in health and experiences is possible and remains a literature gap that needs addressing. However, this was beyond the scope of this study. Ongoing work is examining how the health and experiences of children and young people evolved throughout the pandemic to provide a more comprehensive understanding of how the pandemic impacted their physical, mental, and emotional well-being, as well as their access to health care, education, and social support systems.

### Conclusions

In conclusion, the study used InfraNodus to analyze >8000 free-text responses from children and young people during the COVID-19 pandemic. What emerged was that the majority of the text responses (314/411, 76.4%) indicated negative experiences, and many of the respondents reported experiencing distress across a range of domains related to school, social situations, and mental health. In terms of practical implications for policy makers, educators, and health care providers, this analysis highlights the importance of specific considerations for children and young people when responding to national emergencies; for example, the insights gained show that consistency in relation to education guidance, mitigations for social isolation and loneliness, and increased support on return to education would have reduced much stress; therefore, the application of these measures during and after national emergencies should be considered.

## Appendix

Multimedia Appendix 1 Supplementary text and tables.

## Abbreviations

AI: artificial intelligence
CLoCk: Children and Young People With Long COVID
PCR: polymerase chain reaction

## Data Availability

Children and Young People With Long COVID study data are publicly available in the UK Data Service repository [41].
