# Supplementary material for: Health and Experiences During the COVID-19 Pandemic Among Children and Young People: Analysis of Free-Text Responses From the Children and Young People With Long COVID Study
Source: J Med Internet Res. 2025 Jan 28;27:e63634. doi: 10.2196/63634 (PMC11815313; doi:10.2196/63634)
Supplement: Multimedia Appendix 1 [file jmir_v27i1e63634_app1.docx]

Supplementary Materials

**Text S1.** Application of survey weights developed in Rojas et al. (1) in the present study’s manual sentiment analysis of CYP’s free text responses at enrolment

Survey weights were developed to re-weight from the analytic sample (i.e., those included in the sentiment analysis, *N=*411), to the general population of CYP in England using the same methodology outlined in Rojas et al. (1). The weights developed here accounted for:

1. Not all young people invited to participate enrolling into the study
2. Not all young people enrolling providing a free text response
3. Not all free text respondents having done so relevantly (including within the character limit)
4. Not all those responding relevantly being randomly sampled for manual sentiment analysis

Survey weights were derived as the reciprocal of the following conditional probabilities as shown in Table A below.

First, four ‘mini’ survey weights were calculated for (i) responding given envisioned to take part, (ii) answering free text question given responded, (iii) responding relevantly given answered free text question, and (iv) randomly sampled for sentiment analysis given responded relevantly. Each ‘mini’ survey weight was calculated as the reciprocal of its corresponding conditional probability. These conditional probabilities were computed from logistic regressions, see table below for details.

**Table A.** Variables included in logistic regression models used to produce conditional probabilities for weight generation

| **Conditional Probability** | **Outcome** | **Predictors*** | ***C*-Statistic**** |
| --- | --- | --- | --- |
| Pr(Responding \| Envisioned to take part) | Responding to questionnaire | Sex, Age (cubed and cubed multiplied by log of age), Region, Index COVID-19 Status, IMD (cubed and square rooted) and all 2-way interactions | 0.6118 |
| Pr(Answering free text \| Responded) | Answering free text, question given responded | Sex, Age (cubed and cubed multiplied by log of age), Region, Index COVID-19 Status, IMD (square rooted and to the power of -2), Ethnicity, SWEMWS Score, SDQ Total Score, ED-5D-Y Score, CFS Score, EQ-VAS Score, Loneliness (Self-report), UCLA Loneliness Score, Number of Physical Symptoms, Vaccination Status | 0.5986 |
| Pr(Responding relevantly \| Answered free text) | Responding relevantly (including within character limit), given answered free text question | Sex, Age (Categorical: 11-13; 14-15; 16-17), IMD (squared and linear), Ethnicity, Vaccination Status, EQ-VAS Score, EQ-5D-Y Score, CFS Score | 0.6324 |
| Pr(Randomly sampled \| Responded relevantly) | Randomly sampled for sentient analysis, given responded relevantly | Sex, Age (linear), IMD (squared and linear), CFS Score, Loneliness (Self-report), SDQ Score | 0.5679 |

*All predictors are from time of study enrolment. IMD = Index of Multiple Deprivation; SWEMWS = Short Warwick Edinburgh Mental Wellbeing Scale; SDQ = Strengths and Difficulties Questionnaire; CFS = Chalder Fatigue Scale; EQ-VAS = EuroQol-visual analogue scales; for more information on these predictors see Table 1, Additional File 1 in Rojas et al. (1)

** The predictive performance of the models was assessed using the concordance statistic where 0.8 is strong performance, 0.7 is good and ≤ 0.5 is poor performance (2)


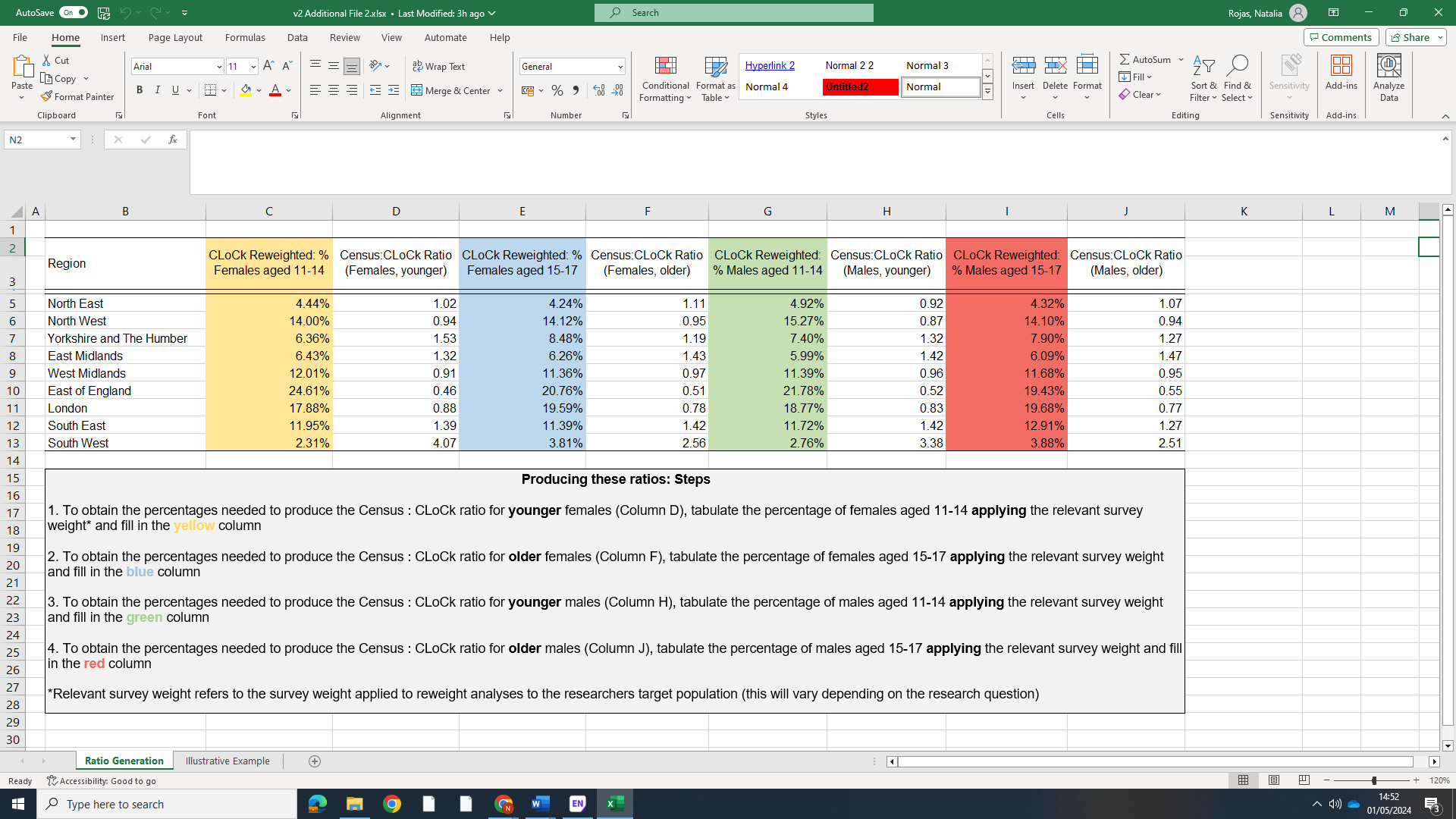
The four ‘mini’ survey weights were combined to obtain a fifth survey weight (‘target population’), which can be used to re-weight from the analytic sample (*N=*411) to the CLoCk target population (i.e., in this case, all those invited to enrol, *N=*219,145). This fifth (‘target population’) survey weight was re-calibrated to the general population of CYP in England by multiplying the target population survey weights by ratios produced using the interactive tool provided in Additional File 2 in Rojas et al. (1) (see screenshot below). For example, the ‘target population’ survey weight for every Female CYP aged 11-14 living in London was multiplied by 0.88, and that for every Male CYP aged 15-17 living in North East England by 1.07, and so on.

The derived ‘general population’ survey weights were trimmed, by reducing extreme weights to a cut-off (median + 3 × interquartile range). Finally, all trimmed ‘general population’ survey weights were re-calibrated back up to the original sum of weights and applied to the sentiment analysis.

**Table S1.** Examples of discrepancies between software and human-assigned sentiment of CYP’s free text responses at enrolment

| **Free text response** | **Researcher assigned sentiment** | **InfraNodus assigned sentiment** |
| --- | --- | --- |
| Downgrading in the work that I produce in school and my attention. As I have lacked a lot in school and feel like the future is closing in on me as I find it hard in school (education & classes) | negative | positive |
| The pandemic hasn't affected my physical health as much as my mental health. Over the past year I've been having issues with my mental well-being and have lacked motivation to do things like taking care of myself, eating etc. | negative | positive |
| The lockdown made me struggle with my mental health at one point to the point I was so anxious I couldn’t eat or leave my house but after a while the doctors put me on [Medication] and it started to get better. I’m doing great now | negative | neutral |
| Isolation and loneliness, missing how things used to be, wishing things weren’t so serious (like future decisions) | negative | positive |

**Table S2**. Demographics of CYP (i) invited, (ii) enrolling, (iii) answering free text question at enrolment, (iv) excluded due to truncation, (v) excluded due to irrelevancy, (vi) included in InfraNodus analysis, and (vii) included in manual sentiment analysis

|  | Invited  (*N=*219,175) | Enrolled  (*N=*31,012) | Answered free text question  (*N=*10,580) | Excluded due to truncation  (*N=*370) | Excluded due to irrelevancy (*N=*1,970) | Included in InfraNodus analysis  (*N=*8,224) | Included in sentiment analysis  (*N=*411) |
| --- | --- | --- | --- | --- | --- | --- | --- |
|  |  |  |  |  |  |  |  |
| COVID-19 status at study invitation |  |  |  |  |  |  |  |
| SARS-CoV-2 Positive | 91,014 (41.53) | 13,690 (44.14) | 4,743 (44.83) | 168 (45.41) | 762 (38.68) | 3,804 (46.25) | 180 (43.80) |
| SARS-CoV-2 Negative | 128,161 (58.47) | 17,322 (55.86) | 5,837 (55.17) | 202 (54.49) | 1,208 (61.32) | 4,420 (53.75) | 231 (56.20) |
|  |  |  |  |  |  |  |  |
| Sex |  |  |  |  |  |  |  |
| Male | 103,939 (47.42) | 11,961 (38.57) | 3,920 (37.05) | 91 (24.59) | 919 (46.65) | 2,905 (35.32) | 141 (34.31) |
| Female | 115,236 (52.58) | 19,051 (61.43) | 6,660 (62.95) | 279 (75.41) | 1,051 (53.35) | 5,219 (64.68) | 270 (65.69) |
| Age at study invitation (Years) |  |  |  |  |  |  |  |
| 11-14 | 112,057 (51.13) | 14,857 (47.91) | 5,184 (49.00) | 117 (31.62) | 1,088 (55.23) | 3,973 (48.31) | 189 (45.99) |
| 15-17 | 107,118 (48.87) | 16,155 (52.09) | 5,396 (51.00) | 253 (68.38) | 882 (44.77) | 4,251 (51.69) | 222 (54.01) |
|  |  |  |  |  |  |  |  |
| Ethnicity |  |  |  |  |  |  |  |
| White | - | 23,198 (74.80) | 7,838 (74.08) | 254 (68.65) | 1,403 (71.22) | 6,167 (74.99) | 308 (74.94) |
| Asian/Asian British | - | 4,553 (14.68) | 1,581 (14.94) | 69 (18.65) | 367 (18.63) | 1,143 (13.90) | 60 (14.60) |
| Black/African/Caribbean/British | - | 933 (3.01) | 343 (3.24) | 10 (2.70) | 69 (3.50) | 264 (3.21) | 18 (4.38) |
| Mixed | - | 1,615 (5.21) | 564 (5.33) | 25 (6.76) | 75 (3.81) | 464 (5.64) | 21 (5.11) |
| Other | - | 524 (1.69) | 187 (1.77) | 8 (2.16) | 44 (2.23) | 135 (1.64) | 1 (0.24) |
| Prefer not to say | - | 189 (0.61) | 67 (0.63) | 4 (1.08) | 12 (0.61) | 51 (0.62) | 3 (0.73) |
|  |  |  |  |  |  |  |  |
| Region (England) |  |  |  |  |  |  |  |
| East Midlands | 14,109 (6.44) | 2,210 (7.13) | 774 (7.32) | 31 (8.38) | 142 (7.21) | 601 (7.31) | 30 (7.30) |
| East of England | 38,901 (17.75) | 6,047 (19.50) | 1,967 (18.59) | 72 (19.46) | 350 (17.77) | 1,543 (18.76) | 63 (15.33) |
| London | 46,300 (21.12) | 6,157 (19.85) | 2,107 (19.91) | 81 (21.89) | 461 (23.40) | 1,565 (19.03) | 72 (17.52) |
| North East England | 8,613 (3.93) | 1,198 (3.86) | 403 (3.81) | 5 (1.35) | 68 (3.45) | 329 (4.00) | 18 (4.38) |
| North West England | 31,289 (14.28) | 3,606 (11.63) | 1,229 (11.62) | 38 (10.27) | 223 (11.32) | 966 (11.75) | 49 (11.92) |
| South East England | 31,567 (14.40) | 4,917 (15.86) | 1,729 (16.34) | 70 (18.92) | 310 (15.74) | 1,347 (16.38) | 80 (19.46) |
| South West England | 8,139 (3.71) | 1,514 (4.88) | 499 (4.72) | 10 (2.70) | 92 (4.67) | 396 (4.82) | 25 (6.08) |
| West Midlands | 22,681 (10.35) | 3,032 (9.78) | 1,046 (9.89) | 38 (10.27) | 184 (9.34) | 822 (10.00) | 37 (9.00) |
| Yorkshire and the Humber | 17,576 (8.02) | 2,331 (7.52) | 826 (7.81) | 25 (6.76) | 140 (7.11) | 655 (7.96) | 37 (9.00) |
| IMD quintile |  |  |  |  |  |  |  |
| 1 (most deprived) | 54,079 (24.67) | 5,345 (17.24) | 1,813 (17.14) | 54 (14.59) | 359 (18.22) | 1,397 (16.99) | 74 (18.00) |
| 2 | 44,757 (20.42) | 5,548 (17.89) | 1,943 (18.36) | 82 (22.16) | 375 (19.04) | 1,484 (18.04) | 80 (19.46) |
| 3 | 39,876 (18.19) | 5,792 (18.68) | 1,985 (18.76) | 54 (14.59) | 367 (18.63) | 1,563 (19.01) | 80 (19.46) |
| 4 | 39,996 (18.25) | 6,656 (21.46) | 2,220 (20.98) | 76 (20.54) | 376 (19.09) | 1,764 (21.45) | 91 (22.14) |
| 5 (least deprived) | 40,467 (18.46) | 7,671 (24.74) | 2,619 (24.75) | 104 (28.11) | 493 (25.03) | 2,016 (24.51) | 86 (20.92) |
|  |  |  |  |  |  |  |  |

*Note.* Data are *n* (%). IMD=Index of Multiple Deprivation

**Table S3.** Associations between sentiment of free text responses provided by CYP at enrolment and their demographic characteristics (N= 411)

|  | **Positive (*N=*57)** | **Neutral (*N=*40)** | **Negative (N=314)** | **p-value^$^** |
| --- | --- | --- | --- | --- |
| COVID-19 Status at index PCR test |  |  |  |  |
| SARS-CoV-2 Positive | 24 (13.33) | 7 (3.89) | 149 (82.78) | 0.001* |
| SARS-CoV-2 Negative | 33 (14.29) | 33 (14.29) | 165 (71.43) |  |
| Sex |  |  |  |  |
| Male | 30 (21.28) | 24 (17.02) | 87 (61.70) | <0.001* |
| Female | 27 (10.00) | 16 (5.93) | 227 (84.07) |  |
| Age at study invitation (Years) |  |  |  |  |
| 11-14 | 22 (11.64) | 23 (12.17) | 144 (76.19) | 0.184 |
| 15-17 | 35 (15.77) | 17 (7.66) | 170 (76.58) |  |
| Ethnicity |  |  |  |  |
| White | 37 (12.01) | 33 (10.71) | 238 (77.27) | 0.493 |
| Asian/Asian British | 11 (18.33) | 6 (10.00) | 43 (71.67) |  |
| Black/African/Caribbean/British | 3 (16.67) | 0 (0.00) | 15 (83.33) |  |
| Mixed | 6 (28.57) | 1 (4.76) | 14 (66.67) |  |
| Other | 0 (0.00) | 0 (0.00) | 1 (100.00) |  |
| Prefer not to say | 0 (0.00) | 0 (0.00) | 3 (100.00) |  |
| Region (England) |  |  |  |  |
| East Midlands | 2 (6.67) | 4 (13.33) | 24 (80.00) | 0.173 |
| East of England | 7 (11.11) | 7 (11.11) | 49 (77.78) |  |
| London | 14 (19.44) | 9 (12.50) | 49 (68.06) |  |
| North East England | 1 (5.56) | 3 (16.67) | 14 (77.78) |  |
| North West England | 2 (4.08) | 3 (6.12) | 44 (89.80) |  |
| South East England | 18 (22.50) | 8 (10.00) | 54 (67.50) |  |
| South West England | 4 (16.00) | 1 (4.00) | 20 (80.00) |  |
| West Midlands | 6 (16.22) | 1 (2.70) | 30 (81.08) |  |
| Yorkshire and the Humber | 3 (8.11) | 4 (10.81) | 30 (81.08) |  |
| IMD quintile |  |  |  |  |
| 1 (most deprived) | 11 (14.86) | 6 (8.11) | 57 (77.03) | 0.191 |
| 2 | 14 (17.50) | 9 (11.25) | 57 (71.25) |  |
| 3 | 5 (6.25) | 8 (10.00) | 67 (83.75) |  |
| 4 | 10 (10.99) | 6 (6.59) | 75 (82.42) |  |
| 5 (least deprived) | 17 (19.77) | 11 (12.79) | 58 (67.44) |  |

**^$^p-value from chi-squared test**

References

1. Rojas NK, De Stavola BL, Norris T, Cortina-Borja M, Nugawela MD, Hargreaves D, et al. Developing survey weights to ensure representativeness in a national, matched cohort study: results from the Children and young people with Long Covid (CLoCk) study. BMC Medical Research Methodology. 2024. doi: 10.1186/s12874-024-02219-0.

2. Hosmer Jr, DW., Lemeshow, S., & Sturdivant, RX. (2013). Applied logistic regression. John Wiley & Sons.
